# Supplementary material for: Online e-learning during the COVID-19 lockdown in Trinidad and Tobago: prevalence and associated factors with ocular complaints among schoolchildren aged 11–19 years
Source: PeerJ. 2022 Jun 6;10:e13334. doi: 10.7717/peerj.13334 (PMC9179615; doi:10.7717/peerj.13334)
Supplement: Supplemental Information 1 [file peerj-10-13334-s001.docx]

**Ocular Health with Digital Device Usage**

This survey aims to investigate the pattern and responsiveness to ocular complaints among students learning remotely during COVID 19 through a quantitative study.

Please note your name will not be used for analysis of results.

1. How old are you? *

- 12-13
- 14-15
- 16-17
- 18-19

1. What is your gender? * Female / Male / Prefer not to say
2. Are you a citizen of Trinidad? * Yes / No
3. What is your ethnicity? *
   - Afro-Trinidadian
   - Indo-Trinidad
   - Chinese
   - Syrian/Lebanese
   - Mixed
   - Other
4. What is the nature of community in which you live? *
   - Rural area
   - Urban area
5. How long have you been living in Trinidad? *
   - Less than 1 year 1-2 years
   - 3-4 years
   - 5 years & above
6. What form are you in? *
   - Form 1
   - Form 2
   - Form 3
   - Form 4
   - Form 5
   - Form 6
7. Did you use a digital device for schooling during COVID 19 Pandemic? * Yes/ No
8. If yes, what Digital Device was used? *
   - Phone
   - Tablet/iPad
   - Laptop/MacBook
   - Desktop
   - Television
   - Other................
9. Before the closure of school due to COVID-19, How many hours a day on average, did you spend on your digital device daily? *
   - Less than an hour 1-2 hours
   - 2-4 hours
   - 4-6 hours
   - More than 6 hours
   - Other: …………………….
10. How many hours a day on average, do you spend on your digital device daily as online teaching is taking place?
    - Less than an hour 1-2 hours
    - 2-4 hours
    - 4-6 hours
    - Other …………………….
11. Do you wear glasses or contact lens?
    - Glasses
    - Contact Lenses
    - Both contact lenses and spectacles
    - Neither glasses nor contact lenses
12. If you do wear glasses or contact lens, do you wear them while using your digital device? *
    - Yes / No/Not applicable
13. What is your preferred position while using your digital device for schooling during COVID 19? * Check all that applies
    - Sitting
    - Standing
    - Laying down
    - Other
14. How long do you spend in that preferred position while using your digital device?
    - Less than 1 hour 1 - 2 hours
    - 2 - 4 hours
    - 4 - 6 hours
    - More than 6 hours
15. Do you have any eye turns? (Eyes turned inward or outward and commonly known as a "lazy eye" or "kokey eye")
    - Inward
    - Outward
    - No
16. Did you experience headaches with use of your device before COVID-19? * Yes / No
17. Did you experience dry/gritty eyes (feeling as though something is in your eyes) with use of your device before COVID-19? * Yes / No
18. Did you experience itchy eyes with use of your device before COVID-19? * Yes / No
19. Did you experience blurry vision with use of your device before COVID-19? * Yes / No
20. Did you experience double vision with use of your device before COVID-19? * Yes / No
21. Do you currently experience headaches with the use of your digital device? * Yes / No
22. Do you currently experience dry/gritty eyes (feeling as though something is in your eyes) with use of your digital device? * Yes / No
23. Do you currently experience itchy with the use of your digital device? * Yes / No
24. Do you currently experience blurry vision with the use of your digital device? * Yes / No
25. Do you currently experience double vision with the use of your digital device? * Yes / No
26. Did you try to resolve any of the symptoms above on your own? * Yes / No
27. If yes, What did you do to resolve the symptoms experienced?
    - …………………………………………………………..
28. When was your last eye exam? *
    - Within the last 6 months
    - Within the last year
    - More than a year ago
    - More than 2 years ago
    - Never had an eye exam
    - Other:
29. If you got an eye exam done within the last year, was it because of some of the complaints mentioned above? * Yes / No / Not applicable
30. What was the nature of the treatment?
    - Glasses
    - Eyedrops
    - Vision Therapy
    - Medication
    - Other: …………………………….

Thank you for participating in this survey

- - ............
