# Supplementary material for: Online e-learning during the COVID-19 lockdown in Trinidad and Tobago: prevalence and associated factors with ocular complaints among schoolchildren aged 11–19 years
Source: PeerJ. 2022 Jun 6;10:e13334. doi: 10.7717/peerj.13334 (PMC9179615; doi:10.7717/peerj.13334)
Supplement: Supplemental Information 2 [file peerj-10-13334-s002.docx]

| Variables | Dry eyes | *Sig.* | Headache | *Sig.* | Blurred vision | *Sig.* |
| --- | --- | --- | --- | --- | --- | --- |
| Socio-demography |  |  |  |  |  |  |
| *Age Category, years* |  |  |  |  |  |  |
| 12-13 | 1.00 |  | 1.00 |  | 1.00 |  |
| 14-15 | 1.99 [1.03, 3.83] | 0.040 | 2.24 [1.20, 4.19] | 0.012 | 1.99[1.02,3.86] | 0.041 |
| 16-17 | 1.67 [0.83, 3.36] | 0.154 | 2.60 [1.30, 5.22] | 0.007 | 2.91[1.39,6.09] | 0.004 |
| 18-19 | 4.73 [2.00, 11.19] | 0.000 | 7.42 [2.58, 21.36] | 0.000 | 3.71[1.51,9.09] | 0.004 |
| *Gender* |  |  |  |  |  |  |
| Boys | 1.00 |  | 1.00 |  | 1.00 |  |
| Girls | 1.66 [1.05, 2.62] | 0.031 | 2.25 [1.39, 3.65] | 0.001 | 2.84[1.71, 4.71] | 0.000 |
| *Nature of community* |  |  |  |  |  |  |
| Rural | 1.00 |  | 1.00 |  | 1.00 |  |
| Urban | 1.20 [0.77, 1.87] | 0.431 | 1.31 [0.82, 2.03] | 0.256 | 1.40 [0.86,2.28] | 0.164 |
| *Citizenship of TnT* |  |  |  |  |  |  |
| Yes | 1.00 |  | 1.00 |  | 1.00 |  |
| No | 0.24[0.05,1.26] | 0.093 | 3.06[0.38,24.51] | 0.291 | 0.53 [0.07,3.83] | 0.531 |
| *Ethnicity* |  |  |  |  |  |  |
| Afro-Trinidad | 1.00 |  | 1.00 |  | 1.00 |  |
| Indo-Tri | 1.55 [0.83,2.90] | 0.173 | 1.20[0.61,2.34] | 0.588 | 1.07[0.54,2.13] | 0.826 |
| Mixed | 1.29 [0.64, 2.59] | 0.476 | 0.78[0.38,1.60] | 0.512 | 0.94[0.45,1.97] | 0.887 |
| Others | 0.99 [0.41, 2.36] | 0.975 | 0.70[0.29,1.71] | 0.444 | 0.30[0.10,0.88] | 0.029 |
| *Year in school* |  |  |  |  |  |  |
| Form 1-3 | 1.00 |  | 1.00 |  | 1.00 |  |
| Form 4-6 | 1.70 [1.09, 2.65] | 0.020 | 1.74[1.08,2.80] | 0.022 | 1.93[1.18,3.16] | 0.008 |
| Average daily hours spent on digital device | |  |  |  |  |  |
| < 2hrs | 1.00 |  | 1.00 |  | 1.00 |  |
| 2-4hrs | 1.91[0.58,6.83] | 0.286 | 1.14[0.40, 3.25] | 0.806 | 1.30 [0.42, 4.03] | 0.645 |
| 4-6hrs | 2.63[1.09,6.35] | 0.032 | 2.08[0.95, 4.55] | 0.066 | 1.36 [0.59, 3.14] | 0.470 |
| >6hrs | 3.24[1.27, 8.27] | 0.014 | 1.90 [0.81, 4.45] | 0.139 | 1.59 [0.63, 4.01] | 0.321 |
| Behavioural Factor: Posture |  |  |  |  |  |  |
| *Lying down* |  |  |  |  |  |  |
| No | 1.00 |  | 1.00 |  | 1.00 |  |
| Yes | 2.91 [1.40, 6.05] | 0.004 | 1.65[0.77,3.55] | 0.196 | 1.54[0.74,3.23] | 0.244 |
| *Sitting* |  |  |  |  |  |  |
| No | 1.00 |  | 1.00 |  | 1.00 |  |
| Yes | 0.52 [0.30, 0.89] | 0.017 | 0.70[0.40,1.21] | 0.207 | 0.74[ 0.43,1.26] | 0.279 |
| *Sitting & Lying down* |  |  |  |  |  |  |
| No | 1.00 |  | 1.00 |  | 1.00 |  |
| Yes | 0.72 [0.34, 1.52] | 0.387 | 1.59[0.67, 3.73] | 0.287 | 1.42[0.63,3.19] | 0.395 |
| Prevention strategies |  |  |  |  |  |  |
| *Wears glasses* |  |  |  |  |  |  |
| No | 1.00 |  | 1.00 |  | 1.00 |  |
| Yes | 1.44 [0.89, 2.32] | 0.133 | 1.03[0.63, 1.68] | 0.901 | 1.00[0.60,1.64] | 0.998 |
| Interventions |  |  |  |  |  |  |
| *Treatment type* |  |  |  |  |  |  |
| Spectacle | 1.00 |  | 1.00 |  | 1.00 |  |
| Medication | 1.25 [0.53, 2.94] | 0.611 | 1.06[0.39,2.80] | 0.905 | 1.13[0.46,2.81] | 0.777 |
| No treatment | 0.44 [0.27, 0.73] | 0.001 | 0.39[0.23,0.68] | 0.001 | 0.51[0.30,0.88] | 0.015 |
| *Eye exam in the last 1 year* |  |  |  |  |  |  |
| No | 1.00 |  | 1.00 |  | 1.00 |  |
| Yes | 8.03 [3.82,16.87] | 0.000 | 6.51[6.74,15.4] | 0.000 | 4.90[2.46,9.78] | 0.000 |
| *Took actions to resolve ocular complaints* | |  |  |  |  |  |
| No | 1.00 |  | 1.00 |  | 1.00 |  |
| Yes | 2.87[1.71, 4.83] | 0.000 | 5.55[2.84, 10.86] | 0.000 | 5.97 [3.00, 11.89] | 0.000 |

^# = any ocular symptom; sig.= significance, set at p<0.05^
