# Supplementary material for: Online e-learning during the COVID-19 lockdown in Trinidad and Tobago: prevalence and associated factors with ocular complaints among schoolchildren aged 11–19 years
Source: PeerJ. 2022 Jun 6;10:e13334. doi: 10.7717/peerj.13334 (PMC9179615; doi:10.7717/peerj.13334)
Supplement: Supplemental Information 3 [file peerj-10-13334-s003.docx]

| Variables | | | Double vision | | *Sig.* | Itchy eyes | | *Sig.* | Any ocular^#^ | |  |
| --- | --- | --- | --- | --- | --- | --- | --- | --- | --- | --- | --- |
| Socio-demography | | |  | |  |  | |  |  | |  |
| *Age Category, years* | | |  | |  |  | |  |  | |  |
| 12-13 | | | 1.00 | |  | 1.00 | |  | 1.00 | |  |
| 14-15 | | | 1.09[0.49,2.42] | | 0.826 | 0.91 [0.49,1.69] | | 0.751 | 1.87 [1.04, 3.63] | | 0.036 |
| 16-17 | | | 1.09[0.472.52] | | 0,835 | 0.96 [0.49,1.88] | | 0.906 | 2.26 [1.16, 4.41] | | 0.017 |
| 18-19 | | | 2.15[0.77,6.02] | | 0.144 | 1.89 [0.87,4.14] | | 0.109 | 10.83 [3.08, 38.16] | | 0.000 |
| *Gender* | | |  | |  |  | |  |  | |  |
| Boys | | | 1.00 | |  | 1.00 | |  | 1.00 | |  |
| Girls | | | 2.03 [1.17,3.53] | | 0.012 | 1.59 [1.03,2.45] | | 0.035 | 1.78 [1.12, 2.82] | | 0.015 |
| *Nature of community* | | |  | |  |  | |  |  | |  |
| Rural | | | 1.00 | |  | 1.00 | |  | 1.00 | |  |
| Urban | | | 1.53 [0.87,2.67] | | 0.134 | 1.26 [0.82,1.95] | | 0.279 | 1.64 [1.04, 2.57] | | 0.032 |
| *Citizenship of TnT* | | |  | |  |  | |  |  | |  |
| Yes | | | 1.00 | |  | 1.00 | |  |  | |  |
| No | | | 0.26[0.03,2.22] | | 0.221 | 0.57 [0.14,2.31] | | 0.432 | 0.52 [0.17, 1.58] | | 0.248 |
| *Ethnicity* | | |  | |  |  | |  |  | |  |
| Afro-Trinidad | | | 1.00 | |  | 1.00 | |  | 1.00 | |  |
| Indo-Tri | | | 1.05[0.51,2.18] | | 0.881 | 1.47[0.78,2.75] | | 0.230 | 1.28 [0.67, 2.43] | | 0.449 |
| Mixed | | | 1.06[0.48,2.34] | | 0.879 | 1.39[0.70,2.77] | | 0.340 | 0.91 [0.45, 1.83] | | 0.792 |
| Others | | | 0.59[0.19,1.81] | | 0.358 | 0.99[0.40,2.40] | | 0.983 | 0.56 [0.24, 1.31] | | 0.185 |
| *Year in school* | | |  | |  |  | |  |  | |  |
| Form 1-3 | | | 1.00 | |  | 1.00 | |  | 1.00 | |  |
| Form 4-6 | | | 1.11[0.65,1.88] | | 0/695 | 1.28[0.83,1.96] | | 0.249 | 2.07 [1.29, 3.32] | | 0.003 |
| Average daily hours  spent on digital device |  |  | |  | | |  |  | |  |  |
| < 2hrs | | | 1.00 | |  | 1.00 | |  | 1.00 | |  |
| 2-4hrs | | | 0.34 [0.09, 1.31] | | 0.116 | 1.33 [0.42, 4.21] | | 0.630 | 0.92 [0.34, 2.46] | | 0.863 |
| 4-6hrs | | | 0.66 [0.27, 1.62] | | 0.367 | 1.90[0.79, 4.61] | | 0.154 | 1.62 [0.75, 3.49] | | 0.221 |
| >6hrs | | | 1.11 [0.43, 2.86] | | 0.825 | 2.17[0.85, 5.52] | | 0.104 | 1.76 [0.75, 4.14] | | 0.195 |
| Behavioural Factor: Posture | | |  | |  |  | |  |  | |  |
| *Lying down* | | |  | |  |  | |  |  | |  |
| No | | | 1.00 | |  | 1.00 | |  | 1.00 | |  |
| Yes | | | 2.95[1.34,6.49] | | 0.007 | 2.80[1.39,5.62] | | 0.004 | 1.38 [0.67, 2.84] | | 0.388 |
| *Sitting* | | |  | |  |  | |  |  | |  |
| No | | | 1.00 | |  | 1.00 | |  | 1.00 | |  |
| Yes | | | 0.50[0.27,0.90] | | 0.023 | 0.64[0.39,1.04] | | 0.074 | 0.81 [0.48, 1.36] | | 0.430 |
| *Sitting & Lying down* | | |  | |  |  | |  |  | |  |
| No | | | 1.00 | |  | 1.00 | |  | 1.00 | |  |
| Yes | | | 0.89[0.38,2.07] | | 0.790 | 0.80[0.40,1.62] | | 0.550 | 1.45[0.65, 3.22] | | 0.361 |
| Prevention strategies | | |  | |  |  | |  |  | |  |
| *Wears glasses* | | |  | |  |  | |  |  | |  |
| No | | | 1.00 | |  | 1.00 | |  |  | |  |
| Yes | | | 0.94[0.53,1.66] | | 0.843 | 1.12[0.72,1.75] | | 0.595 | 1.12 [0.69, 1.80] | |  |
| Interventions | | |  | |  |  | |  |  | |  |
| *Treatment type* | | |  | |  |  | |  |  | |  |
| Spectacle | | | 1.00 | |  | 1.00 | |  |  | |  |
| Medication | | | 1.22[0.46,3.19] | | 0.681 | 2.40[1.06,5.39] | | 0.034 | 0.62 [0.28, 1.39] | | 0.247 |
| No treatment | | | 0.55[0.31, 0.98] | | 0.044 | 0.52[0.32,0.83] | | 0.006 | 0.47 [0.28, 0.78] | | 0.004 |
| *Eye exam in the last 1 year* | | |  | |  |  | |  |  | |  |
| No | | | 1.00 | |  | 1.00 | |  | 1.00 | |  |
| Yes | | | 2.77[1.44,5.35] | | 0.002 | 3.22[1.87,5.55] | | 0.000 | 4.62 [2.16, 9.87] | | 0.000 |
|  |  |  | |  | | |  |  | |  |  |
| No | | | 1.00 | |  |  | |  | 1.00 | |  |
| Yes | | | 3.43 [1.93, 6.09] | | 0.000 | 2.30 [1.45, 3.64] | | 0.000 | 7.36 [3.46, 15.66] | | 0.000 |

^# = any ocular symptom. sig.= significance, set at p<0.05^
